# Reduced Expression of Septin7 Hinders Skeletal Muscle Regeneration

**DOI:** 10.3390/ijms241713536

**Published:** 2023-08-31

**Authors:** László Szabó, Andrea Telek, János Fodor, Nóra Dobrosi, Klaudia Dócs, Zoltán Hegyi, Mónika Gönczi, László Csernoch, Beatrix Dienes

**Affiliations:** 1 Department of Physiology, Faculty of Medicine, University of Debrecen, 4032 Debrecen, Hungary; 2 Doctoral School of Molecular Medicine, Faculty of Medicine, University of Debrecen, 4032 Debrecen, Hungary; 3 ELKH-DE Cell Physiology Research Group, University of Debrecen, 4032 Debrecen, Hungary; 4 Department of Anatomy, Histology and Embryology, Faculty of Medicine, University of Debrecen, 4032 Debrecen, Hungary

**Keywords:** septin7, skeletal muscle, regeneration, central nuclei, muscle injury

## Abstract

Septins are considered the fourth component of the cytoskeleton with the septin7 isoform playing a critical role in the formation of diffusion barriers in phospholipid bilayers and intra- and extracellular scaffolds. While its importance has already been confirmed in different intracellular processes, very little is known about its role in skeletal muscle. Muscle regeneration was studied in a *Sept7* conditional knock-down mouse model to prove the possible role of septin7 in this process. Sterile inflammation in skeletal muscle was induced which was followed by regeneration resulting in the upregulation of septin7 expression. Partial knock-down of *Sept7* resulted in an increased number of inflammatory cells and myofibers containing central nuclei. Taken together, our data suggest that partial knock-down of *Sept7* hinders the kinetics of muscle regeneration, indicating its crucial role in skeletal muscle functions.

## 1. Introduction

Skeletal muscle injury happens quite frequently with different degrees of seriousness. From day-to-day wear and tear stress to complete disruption, the spectrum is very wide. All of them are accompanied by muscle degeneration followed by a highly orchestrated process called muscle regeneration [1]. Complete recovery happens in five consecutive steps: necrosis, inflammation, regeneration, maturation and functional recovery. The initial inflammatory phase is characterized by chemotactic recruitment of circulating leukocytes, starting with neutrophil infiltration, followed by two different subpopulations of macrophages invading the injured muscle [2,3,4]. To restore functional muscle fibers, muscle stem cells (MuSCs), also called satellite cells (SCs), are activated upon muscle injury followed by a myogenic differentiation program to fuse and form new myofibers and to restore muscle tissue [5]. Regeneration can start on day 4 after injury when satellite cells are activated from a dormant stage [2,3,6]. The change in the number of activated satellite cells is a good indicator of muscle regeneration [7]. Satellite cells are localized between the myofiber and basal lamina sequestered in a special microenvironment called the “niche” [8,9]. The basal lamina serves as a scaffold for satellite cells and coordinates their migration after injury [10]. Upon activation caused by a harmful stimulus, quiescent (in G_0_ phase) MuSCs re-enter the cell cycle and undergo asymmetric division. One subpopulation, called quiescent stem cells (QSC), is responsible for self-renewal, providing new stem cells; the other subpopulation is committed to a differentiation program. These subpopulations are different in their expression marker profiles, which helps to characterize the different phases of regeneration. QSCs express the following molecular markers: PAX3, PAX7, CD34, NCAM, c-Met, M-cadherin, syndecan-3 and -4, FoxK1 and VCAM-1 [4,11,12,13,14]. PAX7 is considered the canonical biomarker that coordinates the signalization between the consecutive steps of *de novo* myofiber formation. Subsequently, increased expression of PAX7 indicates regeneration, while a decreasing level of PAX7 shows the completion of skeletal muscle repair [4]. Quiescent stem cells remain dormant to maintain the stem cell pool. On the other hand, activated stem cells preferentially repopulate the SC niche and contribute to long-term muscle regeneration. Activated SCs (ASCs) express PAX7 and MRF (Muscle regulatory factors: MYOD1, MYOG and MYF5) [11,12,13,14]. Oxidative phosphorylation as an energy source in quiescence is switched to glycolysis in proliferating SCs.

Besides alterations in the molecular marker’s expression pattern, morphological changes also accompany muscle regeneration. The first step of satellite cell differentiation is to form myoblasts that further differentiate into myotubes to regenerate fully matured myofibers. One of the major hallmarks of regenerating fibers is the presence of central nuclei. Committed myogenic progenitor cells turn into primary myoblasts to form new myoblasts. Primary myoblasts organize into structures called myotubes and fuse with the injured fiber to repopulate the nuclei lost as a result of injury. Additional myoblasts incorporate into the nascent myotubes, forming new myofibers with increased size and expression of contractile proteins (actin and myosin) [15]. Functional recovery is completed on day 15 with ECM remodeling, revascularization and reestablishment of the neuromuscular junction (NMJ). ECM remodeling is accomplished by the expression of ECM molecules, such as laminin, collagens, proteoglycans and fibronectin [16]. In the final step of skeletal muscle regeneration, satellite cells regulate fibroblast activity that is responsible for ECM synthesis and remodeling [17,18,19].

Septins were first described as regulators of cytokinesis and cell polarity in yeast in 1971 [6]. Since septins interact with actin, microtubules and membrane structures of cells and assemble into filaments, they became generally accepted as the fourth cytoskeletal component besides microfilaments, intermediate filaments and microtubules [20,21,22]. Septin protofilaments are composed of septin hetero-octamers, the “building blocks”, with the core subunits. The number of septin proteins encoded is extremely variable among different organisms. In humans, based on their sequence homology, 13 different isoforms were identified, which were classified into 4 groups (SEPT 2, 3, 6 and 7), with septin7 being the sole member of its group [17]. One septin from each group can form a canonical complex to generate a number of redundant heteromeric complexes [23]. A critical role of septin7 has been proven by different studies [24,25,26]. First, a knockout of septin7 is embryonic lethal, which highlights the importance of this molecule. Second, all septin filaments include septin7. Also, the role of septin7 has been confirmed in different diseases, such as neurological disorders and cancer [22]. The knock-down of septin7 resulted in myofibrillar disorganization, alteration of myosin-heavy chain localization and affected cytoskeleton dynamics [27,28]. In C2C12 cells, an immortalized skeletal muscle cell line, downregulation of septin7 expression resulted in significant changes in cell morphology, decreased cell proliferation and reduced cell differentiation [29]. Inducible reduction in septin7 protein severely modified the muscle architecture in mice. The individual myofibrils became smaller, but their number increased in septin7 KD mice. However, the reduced septin7 expression did not result in an altered excitation–contraction coupling machinery [30]. In a myofiber, the cytoskeleton serves as the structural and supportive scaffold for sarcomeres within the muscle [8]. Skeletal muscle injury destroys the structure of the myofibers, which must be restored by a process called regeneration. All these events (cytokinesis and mitosis) are required for proper muscle regeneration. Therefore, it is plausible to assume that septins participate in muscle regeneration. However, so far there has been no evidence about the role of septins in skeletal muscle regeneration. In our previous report on the role of septin7 in skeletal muscle, based on its role in other cell types and the observation that it is upregulated after injury, we raised the possibility that it could play an important role in muscle regeneration [31]. Here, the role of septin7 in the regeneration process was thus examined using the Cre/Lox system to conditionally downregulate its expression in skeletal muscles of mice. Our findings show that kinetics of regeneration are altered in *Sept7* knock-down animals leading to a prolonged recovery.

## 2. Results

### 2.1. Skeletal Muscle Regeneration Is Hallmarked by Morphological Changes

Since muscle injury impairs the structure of the muscle fiber, it is assumed that its restoration during regeneration involves the cytoskeletal components. Therefore, it is plausible to assume that septins participate in muscle regeneration.

In order to investigate the function of septin7 in skeletal muscle regeneration, we used an animal model previously generated in our lab using Cre−Lox recombination technology. The Cre+Septin-7flox/flox mice (Cre+ in the following) had the MerCreMer construct, while the Cre−Septin-7flox/flox mice (Cre− in the following) did not contain the MerCreMer construct. To assess the role of septin7 in regeneration, mild skeletal muscle inflammation and damage was induced by the injection of 20 µL 1.2% BaCl_2_ to the left *m. tibialis anterior* of the young Cre− and Cre+ mice, while the right m. tibialis anterior served as a non-injected control. This type of injury was used as a simulation of muscle damage that occurs during strenuous muscle work.

**Figure 1:**
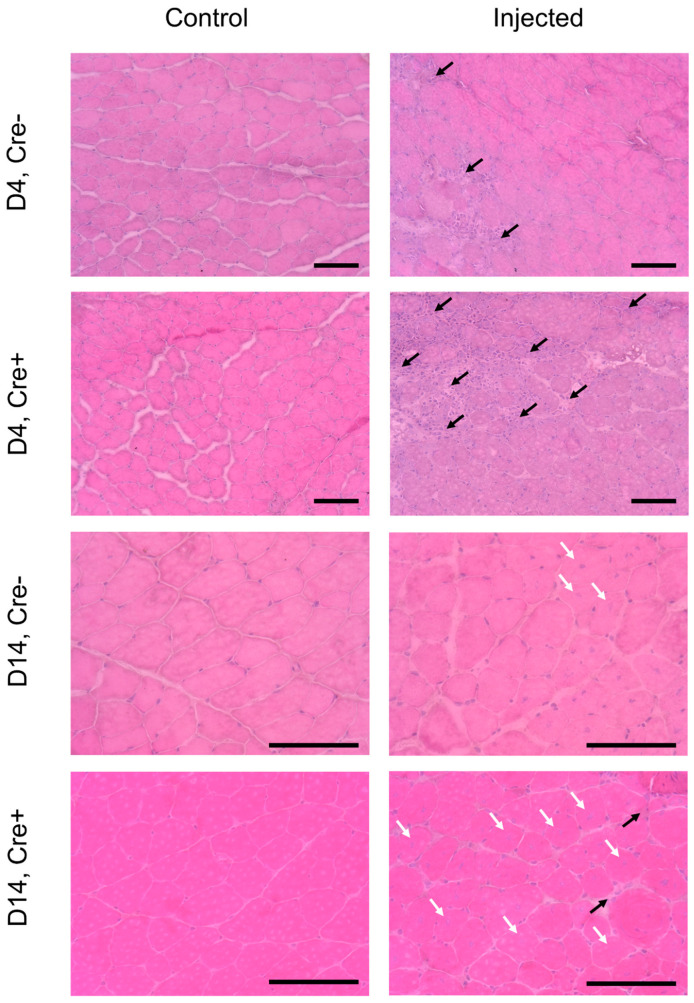
Increased inflammation detected by morphological and histological investigation in *Sept7* conditional knock-down mice. Hematoxylin–eosin staining of a cross section of *m. tibialis anterior* on 4th and 14th days after BaCl_2_ injection into Cre− and Cre+ mice. Control panels indicate non-injected legs. Black arrows indicate inflammatory cells. Scale bar is 100 µm. White arrows point to central nuclei.

Morphological signs of inflammation were visible 4 days after the injection both in the Cre+ and the Cre− mice and were still present 14 days after the injury, as detected by HE staining (Figure 1). The invasion by inflammatory cells (neutrophils, macrophages) of the injected area could be visualized, while non-injected legs did not show any sign of inflammation. Similar changes were detected in BL6/C57 mice (Figure S1).

### 2.2. Skeletal Muscle Injury Induces Septin7 Upregulation

**Figure 2:**
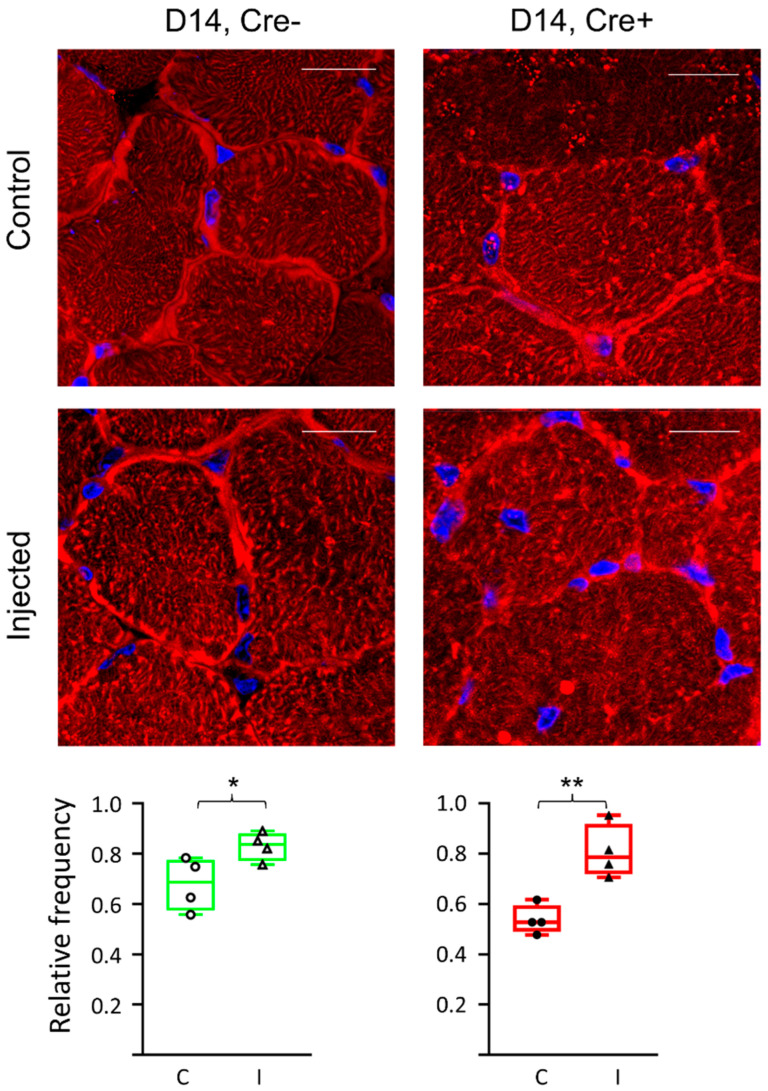
Altered septin structures in regenerating muscle fibers. Fluorescent staining of a cross section of m. tibialis anterior 14 days after BaCl_2_ injection into Cre− and Cre+ mice. Control panels indicate non-injected legs. Septin7 (Cy3) is labelled in red while nuclei are blue (DAPI). Scale bar is 50 µm. Relative frequencies represent the ratio of pixels identified as septin7 signals as compared to total number of pixels on the given image. Relative frequency was counted both in control (C) and injected (I) legs. Green color represents Cre− data and red color refers to Cre+ data. Asterisks indicate statistically significant difference (* *p* < 0.05). Actual *p* values are 0.04 and 0.004 for Cre− (*) and Cre+ (**), respectively.

To study the effect of mild injury on the structure of septin7 filaments, specific immunolabeling was carried out (Figure 2). Using confocal microscopy, a moderate difference was detected in the expression pattern of septin7 filaments between the Cre− and the Cre+ mice. The quantification of the images confirmed that the protein expression increased in the muscle of both the Cre− and the Cre+ mice as a result of the injection (from 67.9 ± 5.2% to 83.0 ± 2.8% and from 53.7 ± 2.9% to 80.8 ± 5.3%, respectively). A significantly lower expression was detected in the muscle sections of the Cre+ animals as compared to the Cre− animals (*p* < 0.05). However, it was not considered sufficient to provide quantitative results based on the microscopic images alone.

To further investigate this structural difference, quantitative analysis was performed both at the mRNA and the protein levels.

**Figure 3:**
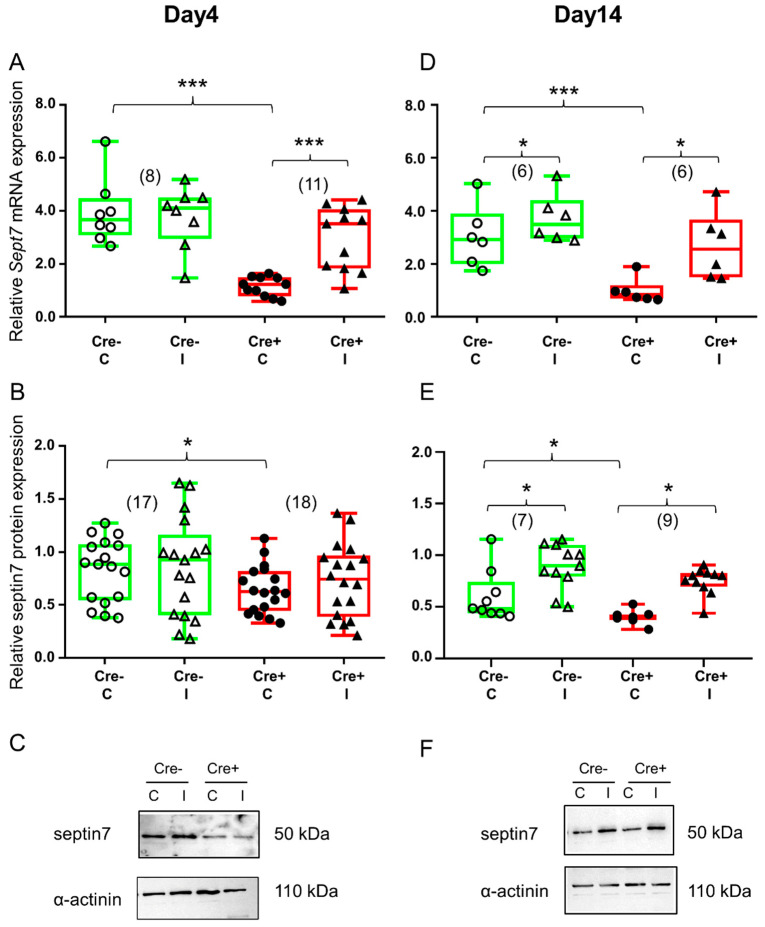
BaCl_2_ injection resulted in elevated septin7 expression. Green color represents Cre− data and red color refers to Cre+ data. Numbers in brackets indicate the number of muscle samples. (**A**) Relative *Sept7* mRNA expression (normalized to AP3D1 as the internal control) in *m. tibialis anterior* from young Cre− and Cre+ mice 4 days after BaCl_2_ injection for control (**C**) and injected (I) legs. (**B**) Relative septin7 protein expression (normalized to α-actinin as the internal control) in m. tibialis anterior from young Cre− and Cre+ mice 4 days after injection. (**C**) Representative images of Western blot showing expression changes in septin7 protein on day 4. (**D**) Relative *Sept7* mRNA expression in m. tibialis anterior from young Cre− and Cre+ mice 14 days after injection. (**E**) Relative septin7 protein expression in m. tibialis anterior from young Cre− and Cre+ mice 14 days after injection. (**F**) Representative images of Western blot showing expression changes in septin7 protein on day 14. Asterisks indicate statistically significant difference (* *p* < 0.05, *** *p* < 0.001 Student’s *t*-test). Actual *p* values when *p* > 0.001 are: (**A**) Cre− C vs. Cre− I *p* = 0.76; (**B**) Cre− C vs. Cre+ C *p* = 0.019; Cre− C vs. Cre− I *p* = 0.75; Cre+ C vs. Cre+ I *p* = 0.62; (**D**) Cre− C vs. Cre− I *p*= 0.010, Cre+ C vs. Cre+ I *p* = 0.017; (**E**) Cre− C vs. Cre+ C *p* = 0.048; Cre− C vs. Cre− I *p* = 0.04; Cre+ C vs. Cre+ I *p* = 0.031.

Skeletal muscle injury did not cause any change in the *Sept7* mRNA level in the Cre− mice during the early stage of regeneration (Figure 3A and Figure S5A). Similarly, no change in *Sept7* mRNA could be detected in the BL6/C57 wild type mice (Figure S2A). On the other hand, in *Sept7* knock-down mice BaCl_2_ injection resulted in a *Sept7* mRNA increase (Figure 3A and Figure S5A) on the 4th day after injection. Western blot analysis revealed partly similar results. Just like *Sept7* mRNA, no difference could be observed in septin7 protein expression in the Cre− mice as a response to injury (Figure 3B and Figure S5B). Interestingly, septin7 protein level was increased in the injected legs of BL6/C57 wild type mice when compared to non-injected legs (Figure S2B). Although *Sept7* mRNA was elevated in the injected muscle of the Cre+ mice, this phenomenon could not be detected at the protein level at this point in time (Figure 3B and Figure S5B).

Ten days later, both mice responded to muscle injury as it was reflected in the changes in septin7 expression. *Sept7* mRNA was increased both in the Cre− and in the Cre+ mice as well as a result of the BaCl_2_ injection (Figure 3D and Figure S5A). Although there was some increase in the *Sept7* mRNA level in BL6/C57 wild type mice, this difference was not significant (Figure S2A). Changes in the septin7 protein supported our mRNA data. Muscle injury caused elevation in septin7 protein both in the Cre− and the Cre+ mice, although the amount of the protein in the Cre+ animal does not reach the values measured in the Cre− even on the 14th day (Figure 3E and Figure S5B). Even though elevation was quite pronounced in BL6/C57 wild type mice as well, it did not reach the level of significance. (Figure S2B).

### 2.3. Upregulation of Transcription Factor PAX7 Accompanies Regeneration

Muscle regeneration is driven by muscle specific stem cells, called satellite cells. Quiescent satellite cells are characterized by the expression of PAX7 with increasing expression upon activation. Therefore, it was plausible to check its expression on the mRNA and protein level after BaCl_2_ injection.

**Figure 4:**
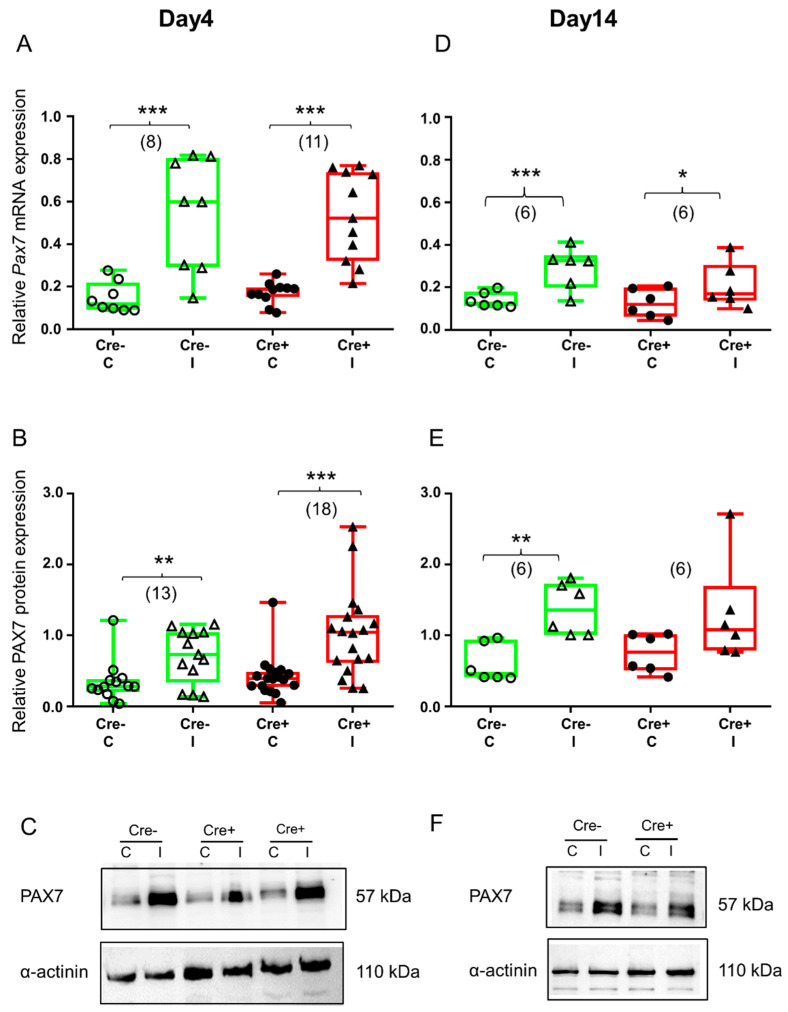
Skeletal muscle injury is reflected in the upregulation of satellite cell marker PAX7. Green color represents Cre− data and red color refers to Cre+ data. Numbers in brackets indicate the number of muscle samples. (**A**) Relative Pax7 mRNA expression (normalized to AP3D1 as the internal control) in *m. tibialis anterior* from young Cre− and Cre+ mice 4 days after BaCl_2_ injection for control (**C**) and injected (I) legs. (**B**) Relative PAX7 protein expression (normalized to α-actinin as the internal control) in *m. tibialis anterior* from young Cre− and Cre+ mice 4 days after injection. (**C**) Representative images of Western blot showing expression changes in PAX7 protein on day 4. (**D**) Relative Pax7 mRNA expression in *m. tibialis anterior* from young Cre− and Cre+ mice 14 days after injection. I Relative PAX7 protein expression in *m. tibialis anterior* from young Cre− and Cre+ mice 14 days after injection. (**F**) Representative images of Western blot showing expression changes in PAX7 protein on day 14. Asterisks indicate statistically significant difference (* *p* < 0.05, ** *p* < 0.005, *** *p* < 0.001 Student’s *t*-test). Actual *p* values when *p* > 0.001 are: (**B**); Cre− C vs. Cre− I *p* = 0.002; (**D**) Cre+ C vs. Cre+ I *p* = 0.010; (**E**) Cre− C vs. Cre− I *p* = 0.002, Cre+ C vs. Cre+ I *p* = 0.086.

As expected, Pax7 mRNA was significantly upregulated 4 days after skeletal muscle injury both in the Cre− mice and the Cre+ mice (Figure 4A and Figure S4A). Results obtained from BL6/C57 wild type mice confirmed the onset of regeneration (Figure S3A). The translation of mRNA was already detectable in the Cre− and the Cre+ mice as reflected in the upregulation of PAX7 protein in injured legs (Figure 4B and Figure S4B). In the injured legs of the BL6/C57 wild type mice, the PAX7 protein was also increased but not to a significant degree (Figure S3B).

By the end of regeneration (day 14), Pax7 mRNA was still elevated in the injected legs of the Cre− and the Cre+ mice (Figure 4D and Figure S4A). The tendency was the same in BL6/C57 wild type mice, but the upregulation was not significant (Figure S3A). Changes in the PAX7 protein confirmed our mRNA data. In the Cre− mice, elevation of the PAX7 protein was still significant (Figure 4E and Figure S4B), just like in the BL6/C57 wild type mice (Figure S3B). However, in the *Sept7* partial knock-down mice, elevation of the PAX7 protein was not significant anymore (Figure 4E and Figure S4B).

### 2.4. Transcription Factor Myogenin Is Upregulated during the Regeneration Phase

After initial regeneration, quiescent satellite cells are turning into differentiating myoblasts. One of the late markers of the myogenic program is transcription factor myogenin, which becomes activated to coordinate the transcription of those proteins that are essential for differentiation. 

**Figure 5:**
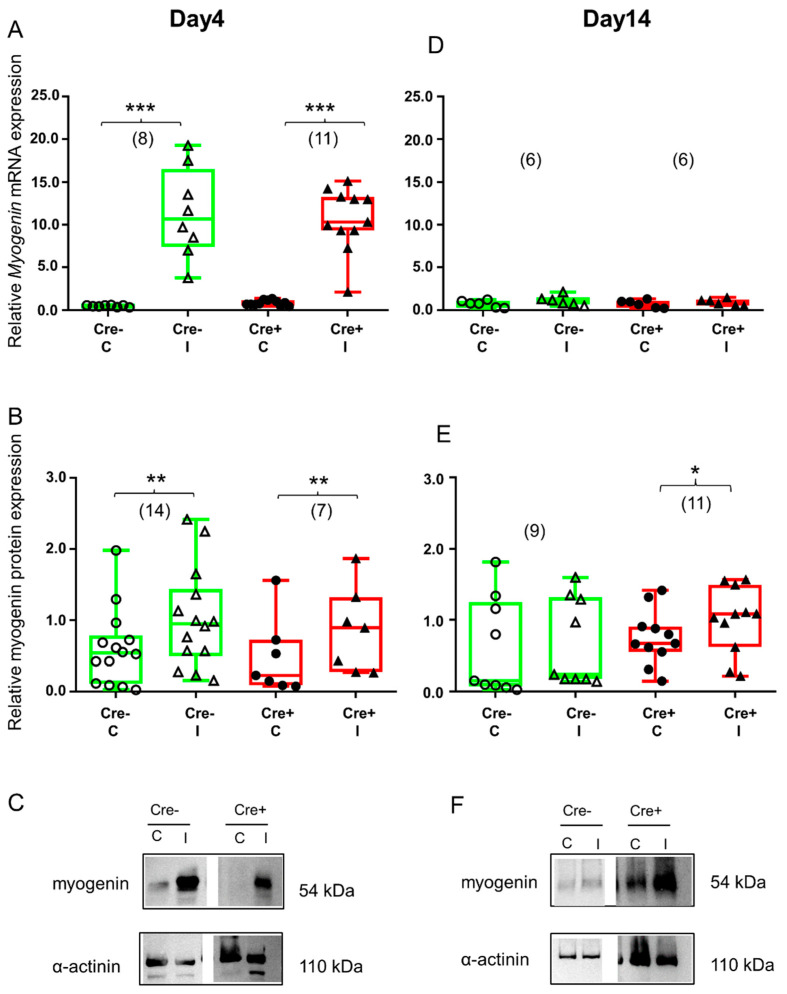
Regeneration marker myogenin is increased during the early stage of regeneration. Green color represents Cre− data and red color refers to Cre+ data. Numbers in brackets indicate the number of muscle samples. (**A**) Relative myogenin mRNA expression (normalized to AP3D1 as the internal control) in *m. tibialis anterior* from young Cre− and Cre+ mice 4 days after BaCl_2_ injection for control (**C**) and injected (I) legs. (**B**) Relative myogenin protein expression (normalized to α-actinin as the internal control) in *m. tibialis anterior* from young Cre− and Cre+ mice 4 days after injection. (**C**) Representative images of Western blot images showing expression changes in myogenin protein on day 4. (**D**) Relative myogenin mRNA expression in *m. tibialis anterior* from young Cre− and Cre+ mice 14 days after injection. (**E**) Relative myogenin protein expression in *m. tibialis anterior* from young Cre− and Cre+ mice 14 days after injection. (**F**) Representative images of Western blot showing expression changes in myogenin protein on day 14. Asterisks indicate statistically significant difference (* *p* < 0.05, ** *p* < 0.005, *** *p* < 0.001 Student’s *t*-test). Actual *p* values when *p* > 0.001 are: (**B**) Cre− C vs. Cre− I *p* = 0.002; Cre+ C vs. Cre+ I *p* = 0.002; (**D**) Cre− C vs. Cre− I *p* = 0.135; Cre+ C vs. Cre+ I *p* = 0.308; (**E**) Cre− C vs. Cre− I *p* = 0.445; Cre+ C vs. Cre+ I *p* = 0.022.

Four days after skeletal muscle injury, myogenin mRNA was already elevated in the Cre− mice (Figure 5A and Figure S4C). This change was confirmed at the protein level as well (Figure 5B and Figure S4D). Similar to the Cre− mice, myogenin mRNA and protein were upregulated as a result of injury in the BL6/C57 wild type mice (Figure S3D,E). Partial downregulation of *Sept7* still resulted in the upregulation of myogenin mRNA (Figure 5A and Figure S4C) as well as protein (Figure 5B and Figure S4D).

By the end of regeneration (Day 14), elevation of myogenin mRNA declined and there was no difference between the control and the injected legs in the Cre− mice (Figure 5D and Figure S4C). This observation was confirmed at the protein level as well (Figure 5E and Figure S4D). The BL6/C57 wild type mice showed the same response to injury (Figure S3D,E). On the other hand, myogenin protein was still upregulated in *Sept7* conditional KD mice (Figure 5E and Figure S4D), indicating the important role of septin7 in this late phase of regeneration. Myogenin mRNA was not elevated anymore (Figure 5D and Figure S4C). 

### 2.5. Differences in ECM Molecule Laminin Expression Reflects Changes in the Final Step of Regeneration

The final step of muscle regeneration is ECM remodeling when ECM molecules, such as laminin, proteoglycan and collagen, are synthetized to provide the proper environment and structural support for the newly formed myofiber. Laminin is a biologically active part of the basement membrane since it promotes differentiation, migration and adhesion. To monitor the final step of recovery, laminin level was detected in both mice 4 and 14 days after the injury.

**Figure 6:**
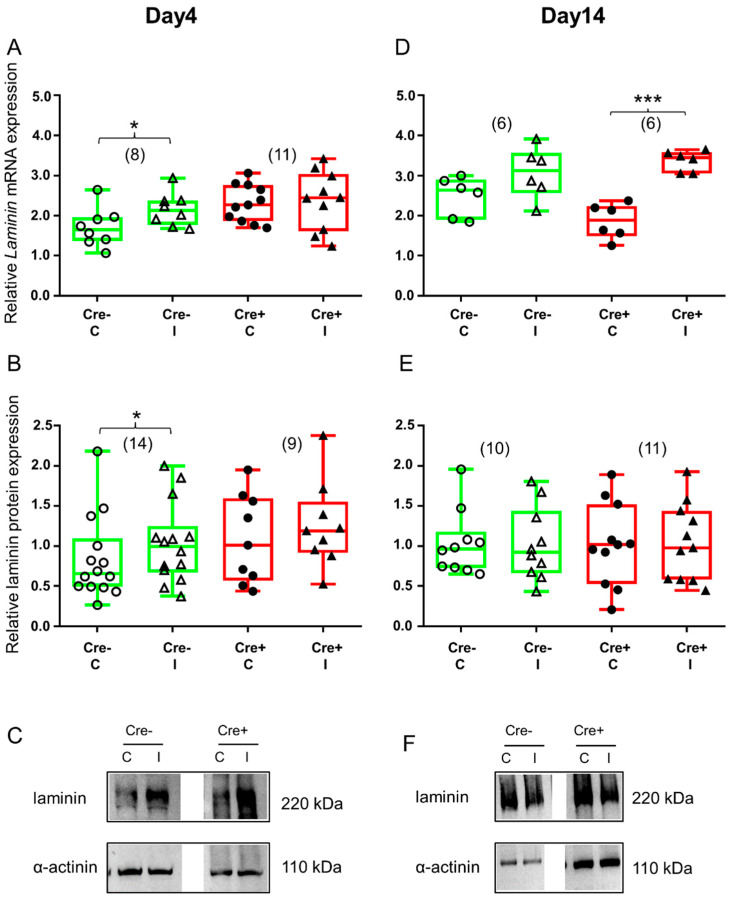
ECM remodeling is different in Cre+ versus Cre− mice as reflected in the changes in laminin expression. Green color represents Cre− data and red color refers to Cre+ data. Numbers in brackets indicate the number of muscle samples. (**A**) Relative laminin mRNA expression (normalized to AP3D1 as the internal control) in m. tibialis anterior from young Cre− and Cre+ mice 4 days after BaCl_2_ injection for control (**C**) and injected (I) legs. (**B**) Relative laminin protein expression (normalized to α-actinin as the internal control) in *m. tibialis anterior* from young Cre− and Cre+ mice 4 days after injection. (**C**) Representative images of Western blot showing expression changes in laminin protein on day 4. (**D**) Relative laminin mRNA expression in m. tibialis anterior from young Cre− and Cre+ mice 14 days after injection. (**E**) Relative laminin protein expression in *m. tibialis anterior* from young Cre− and Cre+ mice 14 days after injection. (**F**) Representative images of Western blot showing expression changes in laminin protein on day 14. Asterisks indicate statistically significant difference (* *p* < 0.05, *** *p* < 0.001 Student’s *t*-test). Actual *p* values when *p* > 0.001 are: (**A**) Cre− C vs. Cre− I *p* = 0.002; Cre+ C vs. Cre+ I *p* = 0.006, Cre+ C vs. Cre+ I *p* = 0.874; (**B**) Cre− C vs. Cre− I *p* = 0.039; Cre+ C vs. Cre+ I *p* = 0.46; (**D**) Cre− C vs. Cre− I *p* = 0.126; (**E**) Cre− C vs. Cre− I *p* = 0.92; Cre+ C vs. Cre+ I *p* = 0.90.

Four days after skeletal muscle injury, the upregulation of both laminin mRNA (Figure 6A and Figure S5C) and protein (Figure 6B and Figure S5D) was detectable in the Cre− mice. Although the tendency was similar in the BL6/C57 wild type mice, upregulation of laminin mRNA and protein was not significant (Figure S2D,E). However, there was no difference between the injured and non-injured legs of Cre+ mice either in the mRNA level (Figure 6A and Figure S5C) or in the protein expression (Figure 6B and Figure S5D).

By the end of regeneration, the upregulation of laminin mRNA was no longer detectable in the Cre− mice (Figure 6D and Figure S5C). A Western blot analysis of the laminin protein confirmed mRNA data (Figure 6E and Figure S5D). Similarly, in the BL6/C57 wild type mice, muscle injury did not cause expression change either in laminin mRNA (Figure S2D) or in protein (Figure S2E). On the other hand, laminin mRNA was still elevated in the injured legs of the Cre+ mice (Figure 6D and Figure S5C). Although laminin protein was also higher in the injured legs of the Cre+ mice when compared to non-injured legs, this difference was not statistically significant (Figure 6E and Figure S5D).

### 2.6. Partial Knock-Down of Sept7 Delays Regeneration as Reflected in Morphological Changes

Our data indicated the contribution of septin7 in muscle regeneration, namely partial knock-down of *Sept7* resulted in differences in the expression of molecular markers accompanying regeneration. The central nuclei in skeletal muscles indicate integration of newly differentiated SC-originated myoblasts into the damaged muscle fibers. In the final step, nuclei regain their original subsarcolemmal location.

**Figure 7:**
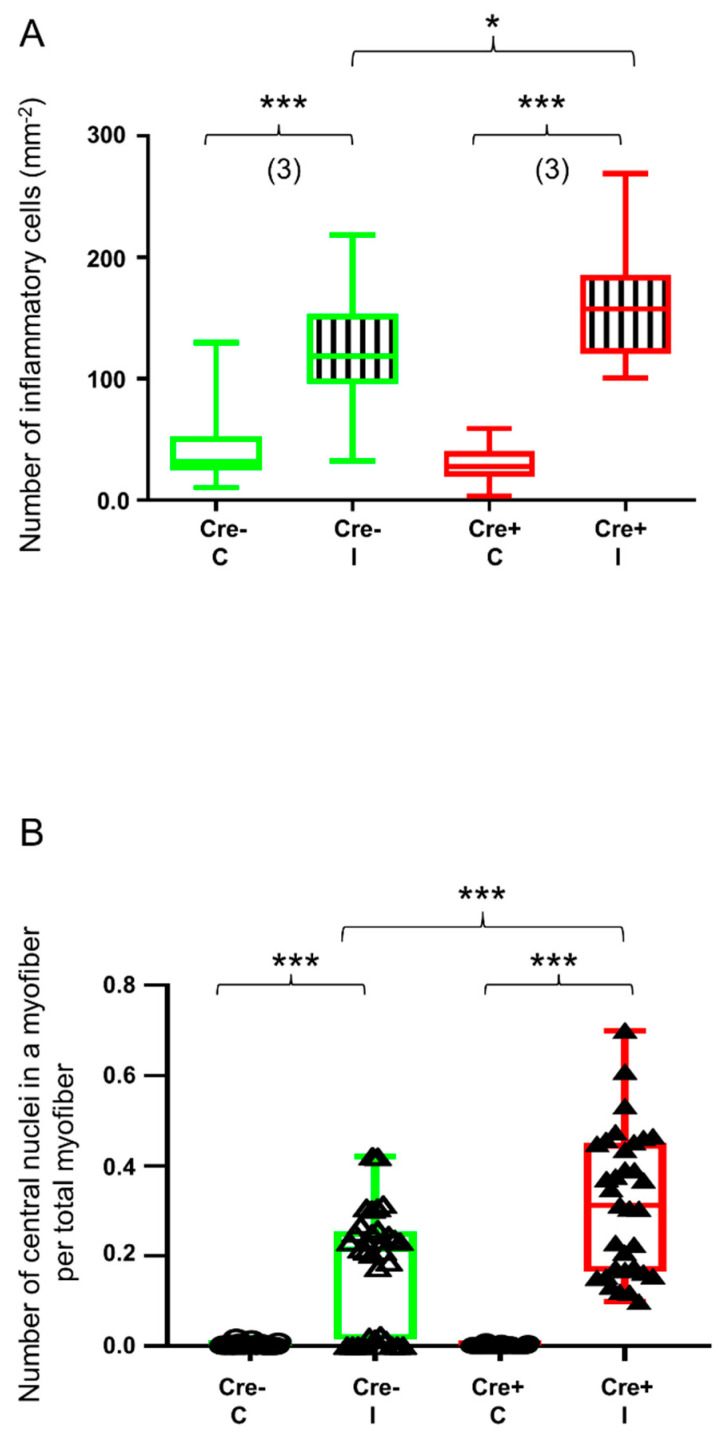
Quantitative histological analysis shows delayed kinetics of regeneration in septin7 downregulated mice. Green color represents Cre− data and red color refers to Cre+ data. Numbers in brackets indicate the number of muscle samples. (**A**) Number of inflammatory cells per mm^2^. Inflammatory cells were counted on HE sections from Cre− and Cre+ mice 14 days after BaCl_2_ injection. Ten images were taken from one section, number of sections = 3 from all groups. (**B**) Number of centrally located nuclei in regenerating myofibers per total number of myofibers 14 days after injection. Asterisks indicate statistically significant difference (* *p* < 0.05, *** *p* < 0.001 Student’s *t*-test). Actual *p* value when *p* > 0.001 is (**A**) *p* = 0.024.

To further investigate the role of septin7 in muscle regeneration, we performed morphological analysis. The number of inflammatory cells and the number of myofibers containing central nuclei were determined 14 days after the injury using the HE-stained sections presented in Figure 1. To confirm the invasion by inflammatory leukocytes, fluorescent immunostaining was performed against CD45, the leukocyte common antigen (Figure S7). Although the BaCl_2_ injection resulted in significant upregulation of inflammatory cells both in the Cre+ and the Cre− mice, the degree of upregulation was significantly higher in the Cre+ mice when compared to the Cre− mice (Figure 7A). Similarly, the number of myofibers containing central nuclei was significantly higher in the Cre+ mice (Figure 7B and Figure S6B) versus the Cre− mice (Figure 7B and Figure S6A), while the central nuclei could hardly be identified in the non-injected mice (Figure 7B).

## 3. Discussion

Muscle regeneration following skeletal muscle damage is a precisely synchronized process that is essentially due to the activation of muscle resident satellite cells (MuSCs) located in a unique microenvironment known as the niche. As a result of a stimulus (injury, exercise, inflammation, etc.), quiescent satellite cells are activated, resulting in the formation of myoblasts, which, after proliferation, fuse with one another or with existing myofibrils, leading to muscle regeneration [32]. In addition to the activation of satellite cells, several myogenic regulatory factors and a permanent cooperation between MuSCs and their niche components are essential during this very complex regeneration process [33,34,35,36]. Besides epigenetic factors, the state of the cytoskeletal complex also plays a significant role in maintaining the regenerative capacity of stem cells [37,38]. The lack of dystrophin has been shown to have a serious consequence from the point of view of regeneration in dystrophic muscles: it leads to a decrease in the self-renewal capacity of stem cells during the regenerative response [39]. During the study of the deteriorating muscle regeneration capacity associated with aging, the modification of the actin cytoskeleton was also described as a consequence of the decreasing expression of fibronectin [40].

Recently, the activation of a self-repair mechanism independent of satellite cells has also been described [41]. Although the signaling pathway activated in this mechanism does not affect satellite cells, the importance of the cytoskeletal structure, namely microtubules and dynein, is clearly demonstrated. However, the mapping of the role of the cytoskeletal system in repair mechanisms is still far from complete.

Since their identification in 1970s, the role of septins has been studied in detail in many cell physiological processes and in many cell types, but not much is known about their role in muscle [42,43]. Our group recently published its results on the importance of septin7 in muscle development and function [31]. In this work, there were implications that the expression of septin7 might be related to muscle regeneration. Here we demonstrated that septin7 significantly contributes to the healthy recovery of muscle after injury.

To study its role, a mouse model with skeletal muscle-specific knock-down of the *Sept7* gene using the Cre/Lox system was generated in our laboratory. In this mouse model, the expression of *Sept7* in skeletal muscle fibers was decreased by approximately 40% (knockout of *Sept7* is embryonic lethal). Muscle atrophy and reduced muscle tone were observed in the genetically modified mice [31]. This impaired morphology and function raises the question of whether reduced septin7 expression, which appears to be critical during muscle development, is relevant during adult muscle regeneration.

During the assessment of the role of *Sept7* in this latter process, measurements were performed on the Cre+Septin-7^flox/flox^ mice (Cre+) and the Cre−Septin-7^flox/flox^ mice (Cre−), and the BL6/C57 mice were used as absolute control.

The invasion by inflammatory cells was clearly visible in each mouse strain 4 days after the BaCl_2_ injection, a well-known approach to induce mild chemical injury. Barium ions can affect cells in two different ways. Barium ions can block potassium channels and alter the membrane potential, leading to a terminal depolarization. Barium can also activate apoptotic proteins. Any of these methods can cause cell damage; however, this phenomenon is more complex in a multinucleated muscle fiber than in other cell types. Fibers can be partially damaged or destroyed because they contain their genetic code in multiple copies.

Confocal images revealed some differences in the septin7 expression pattern between the Cre+ and the Cre− mice, which was reflected in the changes of protein and mRNA levels as well. The amount of Sept7 increased in both animals (Cre−, Cre+) as a result of injection; however, these changes were more pronounced in the Cre+ mice. At the mRNA level, this elevation was already significant in the Cre+ animal on the 4th day (injected leg normalized to non-injected leg), while at the protein level it was visible on the 14th day. As expected, the amount of mRNA in the control leg of the Cre+ animal was also significantly lower in absolute value than in the Cre− animal. After regeneration was triggered, the increase in mRNA started in the Cre+ mouse quickly, helping to produce the amount of septin7 necessary for regeneration. The increase in the amount of protein occurred with a delay of a few days.

The coordination of the consecutive steps required for proper muscle regeneration activation of transcription factors is indispensable. PAX7, the master regulator of the regeneration phase, controls the different steps and signalization pathways. Interestingly, no significant difference in the PAX7 protein and the mRNA level could be observed between the control legs of the Cre− and the Cre+ mice. It can be assumed that the regenerative capacity of the skeletal muscles of the two animals is initially similar, since PAX7, expressed by satellite cells and required for muscle regeneration, is almost equally available. The fact that the Cre recombinase gene was driven by the human alpha-skeletal actin (ACTA1) promoter probably also contributed to this, which is also a limitation of our experiments; hence, *Sept7* expression only decreased in myofibers. Since PAX7-positive satellite cells express a different type of actin, the gene modification did not affect these cells, so no expression changes occurred in them.

The induction of injury increased the level of PAX7 in all cases, indicating the initiation of the regeneration cascade. Myogenin, another transcription factor that plays a significant role in the terminal differentiation of myogenic progenitor cells, partially reflects a similar expression. In the resting state (control leg), we see no difference between the Cre+ and the Cre− animals either at the mRNA or at the protein level.

On the other hand, the elevated myogenin protein expression resulting from the injection of BaCl_2_ persists even on day 14 in the Cre+ animal, indicating a change in the kinetics of regeneration and prolonged myofiber formation as compared to the Cre− mice, where the earlier higher expression was no longer clearly detectable by the 14th day. This is not the only sign of an alteration in the kinetics of recovery and final remodeling due to partial knock-down of *Sept7*. This was also reflected in the expression changes in the ECM molecule laminin that was significantly upregulated 4 days after the injury in the Cre− mice, whereas partial knock-down of *Sept7* hindered upregulation. In the final stage of remodeling, however, only the Cre+ mice responded with the increase in laminin mRNA. The increase in the amount of protein presumably occurs with a slight delay, after the 14th day. This shift in the Laminin expression could be due to partial knock-down of septin7 by the altered cytoskeleton. 

**Figure 8:**
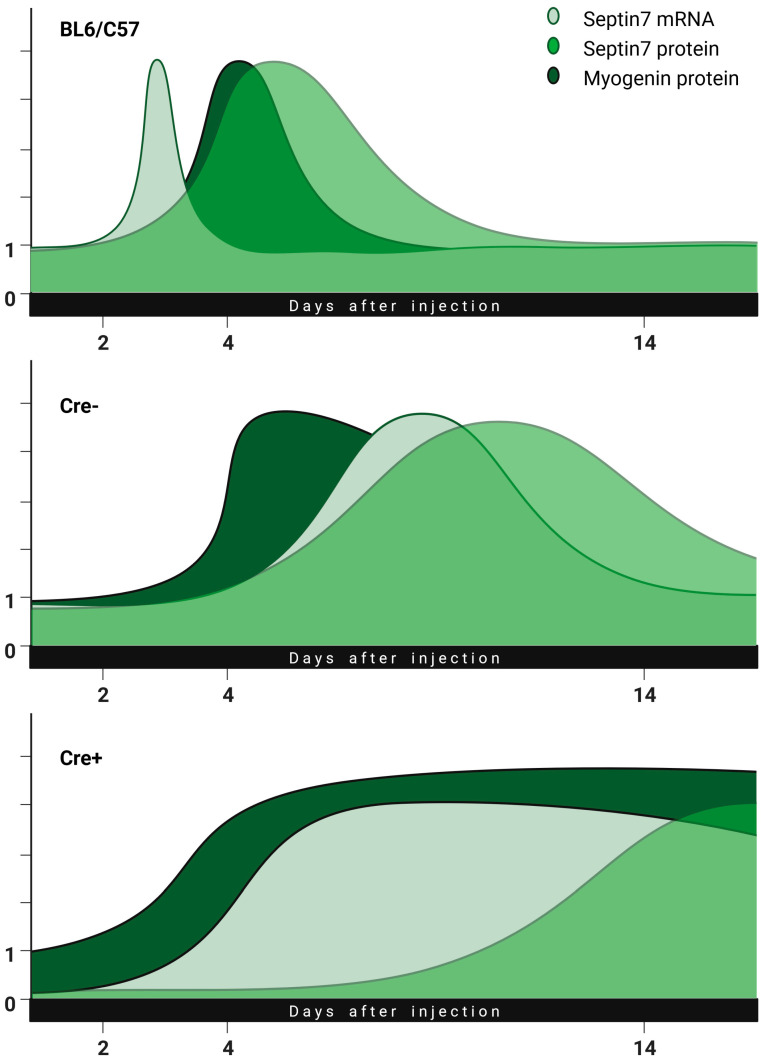
Illustration of prolonged regeneration due to reduced expression of septin7. In *Sept7* knock-down animals, the regeneration lasts longer in parallel with delayed increase in septin7 expression as compared to BL6/C57 or Cre− animals (biorender.org, accessed on 23 March 2023).

Changes in the regeneration process are illustrated in Figure 8. This combined hypothetical summary figure merges the previous data found in the literature with our idea, to which our measurement points fit well. Changes in the amounts of septin7 mRNA, septin7 protein and myogenin were illustrated as a function of regeneration time in the BL6/C57, Cre− and Cre+ mice. Based on our experiments, we believe that the originally reduced septin7 expression significantly prolongs the regeneration phase. The injury led to an increase in the amount of septin7 in all animals, suggesting that the protein is necessary for regeneration. The use of tamoxifen alone resulted in a slight delay in septin7 production but did not modify its dynamics (a slight shift to the right in the curves for Cre− as compared to BL6/C57 animals). At the same time, the transcription of a sufficient amount of septin7 protein from mRNA happens more slowly in the Cre+ animals while the level of myogenin remains high. It is important to note here that the amount of *Sept7* mRNA in the Cre+ animals increased to the point seen in the Cre− mice despite the presence of the floxed gene, indicating that a given amount of septin7 in indispensable for the process. From this, we might conclude that terminal differentiation of myotubes does not take place in the absence of septin7. In this framework, regeneration is not completed until the required amount of septin7 protein is produced, which is indicated by a long-lasting levated myogenin level. The delayed expression of laminin protein in Cre+ animals is also consistent with this hypothesis.

A striking difference was also observed in the morphological changes accompanying the regeneration. The number of inflammatory cells was increased as a result of mild injury; however, the degree of increase was significantly higher in the Cre+ mice, indicating that the partial knock-down of *Sept7* delays the recovery. Central nuclei are potent indicators of muscle regeneration, indicating the ongoing protein synthesis to fulfill the needs of the affected segments of the myofibers. The number of myofibers containing central nuclei is also significantly higher in the Cre+ mice versus the Cre−, further highlighting the role of septin7 in muscle repair.

Several studies pointed out the role of actin cytoskeleton remodeling as well as the significance of microtubule dynamics in myotube formation [44,45,46,47,48]. Here, we show that another element of the cytoskeletal system, septin, might also be essential for this process. Although the downregulation of septin7 protein in this model is not particularly pronounced, the changes observed are significant despite their small magnitude. In the case of a reduced amount of septin7, the formation of skeletal muscle slows down significantly or may not even occur completely. However, further experiments are needed to confirm the exact role of this protein in the recovery of skeletal muscle following injury.

## 4. Materials and Methods

### 4.1. Animal Care

Animal experiments followed the guidelines of the European Community (86/609/EEC). The experimental protocol was approved by the institutional Animal Care Committee of the University of Debrecen (2/2019/DEMAB). The mice were housed in plastic cages with mesh covers and fed with pelleted mouse chow and water ad libitum. Room illumination was an automated cycle of 12 h light and 12 h dark, and room temperature was maintained within the range of 22–25 °C. 

To assess the function of Sept7 in skeletal muscle, a mouse model with skeletal muscle specific knock-down of *Sept7* gene using the Cre/Lox system was generated. Briefly, HSA-Cre79 transgenic mice have the cre recombinase gene driven by the human alpha-skeletal actin (HSA or ACTA1) promoter. When bred with mice containing a loxP-flanked sequence of exon 4 encoding the GTP-binding P-loop of *Sept7* in the mouse genome, Cre−mediated recombination will catalyze exon 4-excision resulting in an additional frame shift mutation downstream to this exon. The Cre+Septin-7flox/flox mice (Cre+ in the following) had the MerCreMer construct, while the Cre−Septin-7flox/flox mice (Cre− in the following) did not contain the MerCreMer construct. The Cre+ hemizygous mice and their Cre− homozygous littermates following 3 months of Tamoxifen feeding were selected for the experiments.

### 4.2. Muscle Regeneration

Skeletal muscle injury was accomplished via a BaCl_2_ injection. A quantity of 20 µL of 1.2% BaCl_2_ (dissolved in physiological saline) was injected to the left m. tibialis anterior muscle (TA) of the BL6/C57, the Cre− and the Cre+ mice (right m. tibialis anterior muscle was the non-treated control). The mice were sacrificed 4 and 14 days later followed by removal of TA. 

### 4.3. Experimental Design

**Figure 9:**
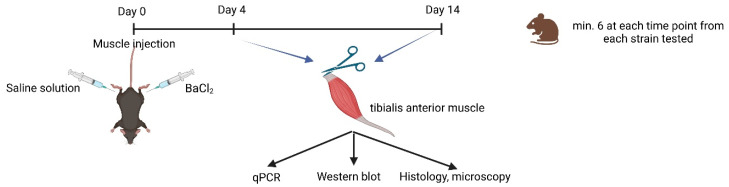
Illustration of experimental design (biorender.org, accessed on 23 March 2023).

The experimental design is presented in Figure 9. Samples were obtained from both injected and non-injected muscles for mRNA and protein expression measurement and for cryosection/paraffin-embedded sections. Histological sections were stained with hematoxylin and eosin (HE). Hematoxylin stains cell nuclei a purplish blue, and eosin stains the extracellular matrix and cytoplasm pink. Each data point on the graphs (Figure 2, Figure 3, Figure 4, Figure 5 and Figure 6) represent one individual mouse. The numbers in brackets represent the number of animals. The number of samples was chosen to be at least 6 per statistical groups.

### 4.4. Western Blotting

M. tibialis anterior samples were homogenized in a lysis buffer (20 mM Tris–HCl, 5 mM EGTA), Protease Inhibitor Cocktail (Sigma, St. Louis, MO, USA) with HT Mini homogenizer (OPS Diagnostics USA, Lebanon, NJ, USA). Fivefold concentrated electrophoresis sample buffer (20 mM Tris–HCl, pH 7.4, 0.01% bromophenol blue dissolved in 10% SDS, 100 mM β-mercaptoethanol) was added to total lysates to adjust equal protein concentration of samples and boiled for 5 min at 90 °C. A quantity of 10 μg of total protein were loaded to each lane and separated in 7.5% SDS–polyacrylamide gel. Proteins were transferred to nitrocellulose membranes and blocked with 5% non-fat milk dissolved in phosphate saline buffer (PBS). Membranes were incubated with primary antibodies overnight at 4 °C (see Key resources table). Membranes were washed for 30 min in PBS supplemented with 1% Tween-20 (PBST), followed by incubation with HRP-conjugated secondary antibodies (see Key resources table). Signals were detected using enhanced chemiluminescence (Thermo Fisher Scientific, Waltham, MA, USA). The optical density of signals was measured by ImageJ (v1.52a) software (NIH, Bethesda, MD, USA) and results were normalized to the optical density of proteins used as loading control.

### 4.5. RNA Preparation, RT-PCR, and Quantitative Real-Time PCR

#### 4.5.1. RNA Isolation

The total RNA fraction was isolated with TRI reagent (MRC, cat. no.: NR118) from homogenized m. tibialis anterior (TA) skeletal muscle specimens. The isolated RNA was resuspended in nuclease-free water (NFW) and stored at −80 °C temperature. The RNA concentration and quality were determined by a spectrophotometer at 260 nm wavelength. (NanoDrop ND1000; Promega Biosciences, Madison, WI, USA). The isolated RNA was treated with DNase and RNase inhibitor (Ambion, Austin, TX, USA).

#### 4.5.2. Reverse Transcription (RT) and Quantitative Polymerase Chain Reaction (qPCR)

A quantity of 1000 ng of the isolated total RNAs was reverse transcribed into complementary DNA (cDNA) with Omniscript RT kit (Qiagen, cat. no.: 205113); cDNA synthesis was completed using random hexamers in a 25 µL reaction volume.

For quantitative RT-PCR, Taqman Gene Expression Assays were used with the Taqman™ Gene Expression Master Mix (Applied Biosystems, Foster City, CA, USA). The amplification was performed using a Light Cycler 480 Master instrument (Roche) (cat. no. for plates, Roche: 04729692001; cat. no. for sealing foils, Roche: 04729757001). Mouse Taqman gene expression assays were purchased from Thermo Fisher Scientific (Waltham, MA, USA), Pax7 (Mm01354484_m1), Sept7 (Mm00550197_m1), Myogenin (Mm00446194_m1) and Laminin (Mm00550083_m1).

The amplification program was 10 min at 95 °C followed by 50 cycles of 15 s at 95 °C and 1 min at 60 °C. The relative expression values for each transcript of interest were calculated by the comparative Ct method, and Ap3d1 (Mm00475961_m1) was used for normalization.

#### 4.5.3. Evaluation of qPCR Data

All qPCR reactions were conducted in triplicates. The C_p_ values were determined with the Light Cycler 480 SW 1.5.0 software (Roche). The relative copy numbers were calculated via the ∆C_p_ method. The ratios of the values of the examined and normalization genes gave the relative expression levels.

### 4.6. Immunolabeling and Confocal Imaging

The m. tibialis anterior samples were obtained and immediately embedded into Cryomatrix (6769006, Thermo Fisher Scientific, Rockford, IL, USA). The cross-sections of m. tibialis anterior that were 7 µM thick were placed onto Superfrost Ultra Plus glass slides (Thermo Fisher Scientific, Rockford, IL, USA) and stored at −20 °C. The slides were thawed at room temperature for 15 min and fixed in 4% PFA for 15 min. The samples were washed three times with 100 mM glicine-PBS solution for 15 min on 22 °C to neutralize excess PFA. Triton-X-100 (0.5 *v*/*v*%, 10 min, 22 °C) was used for permeabilization. The non-specific biding sites were blocked with Carbo-Free Blocking Solution (SP-5040, VECTOR Laboratories, Burlingame, CA, USA). Anti-septin7 and anti-CD45 primary antibody (see Key resources table) were applied overnight at 4 °C. This was followed by a labelling with Cy3 conjugated anti-rabbit secondary antibody (A10520, Life technologies, Eugene, OR, USA) for 60 min. Autofluorescence was reduced with VECTOR TrueVIEW Autofluorescence Quenching kit (SP-8500, VECTOR Laboratories, Burlingame, CA, USA). Mounting was completed with VECTASHIELD Antifade Mounting Medium with DAPI (H-1200-10, VECTOR Laboratories, Burlingame, CA, USA). Confocal images were taken with Zeiss laser scanning confocal microscope (Zeiss LSM880 Airyscan; Zeiss, Oberkochen, Germany) using 405 and 543 nm excitation wavelengths using a 63× oil immersion objective. During the quantification of the images, the background was determined and the pixels with higher intensity were interpreted as the expression of septin7 protein. The ratio of these pixels in relation to the total number of pixels of the given image was calculated. The relative frequencies thus obtained were compared.

### 4.7. Statistical Analysis

The pooled data were expressed as mean ± standard error of the mean (SEM). The differences between the control mice and animals on the tamoxifen diet were assessed using one-way analysis of variance (ANOVA) and the all pairwise Bonferroni’s multiple comparison method using the statistical program Prism (GraphPad Software v8.0.1, San Diego, CA, USA). A paired T-test was used to compare the control and injected muscles and a *p* value of less than 0.05 was considered statistically significant. The normal distribution of the data was tested using the Shapiro–Wilk test (GraphPad Software v8.0.1, San Diego, CA, USA).

## Data Availability

Not applicable.

## Supplementary Materials

The following supporting information can be downloaded at https://www.mdpi.com/article/10.3390/ijms241713536/s1.
