# Supplementary material for: Reduced Expression of Septin7 Hinders Skeletal Muscle Regeneration
Source: Int J Mol Sci. 2023 Aug 31;24(17):13536. doi: 10.3390/ijms241713536 (PMC10487768; doi:10.3390/ijms241713536)
Supplement: Supplementary file 1 [file ijms-24-13536-s001.zip › ijms-2462621-supplementary.pptx]

## Slide 1
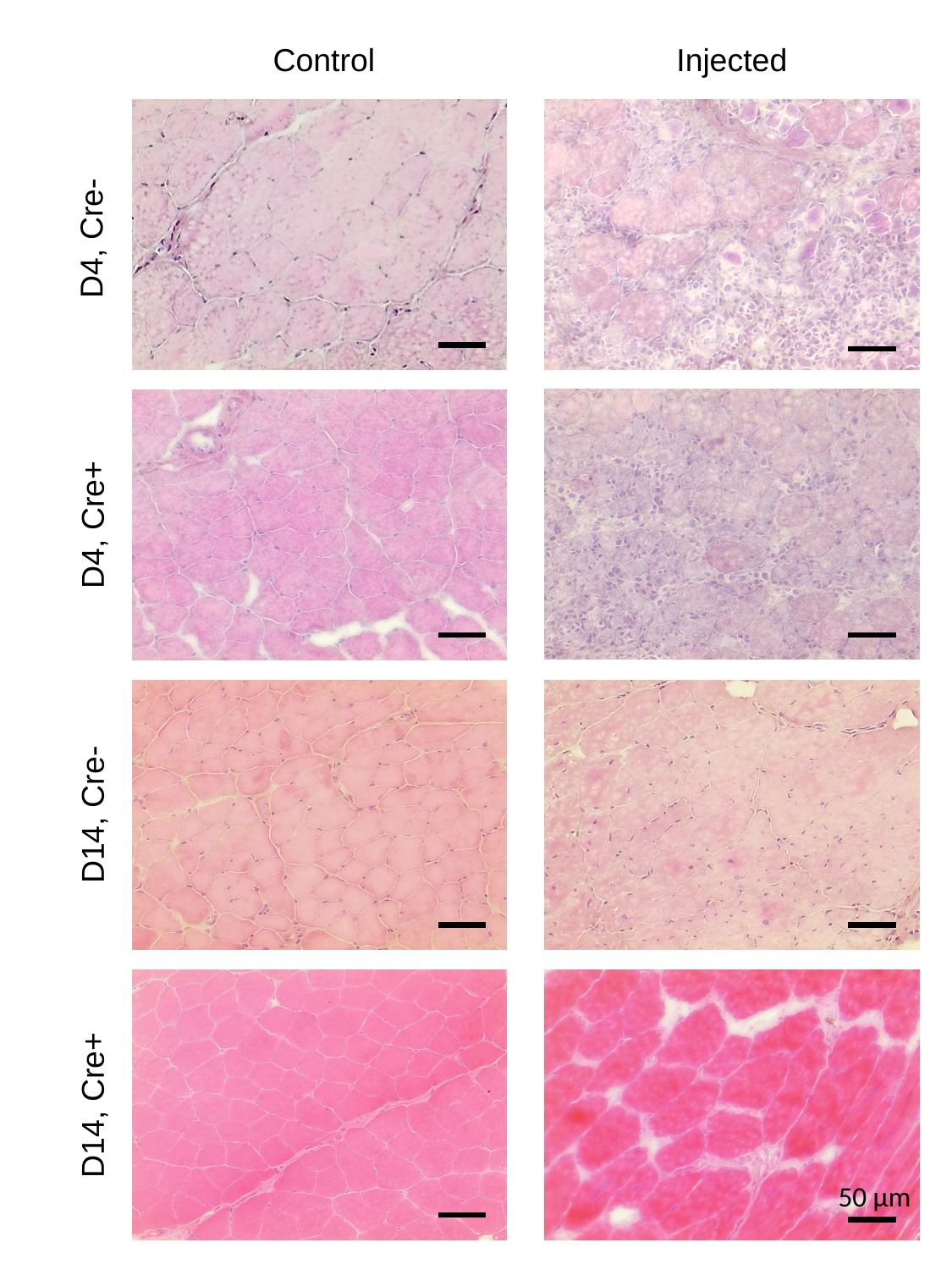

Control
Injected
D4, Cre-
D4, Cre+
D14, Cre-
D14, Cre+
50 μm

## Slide 2
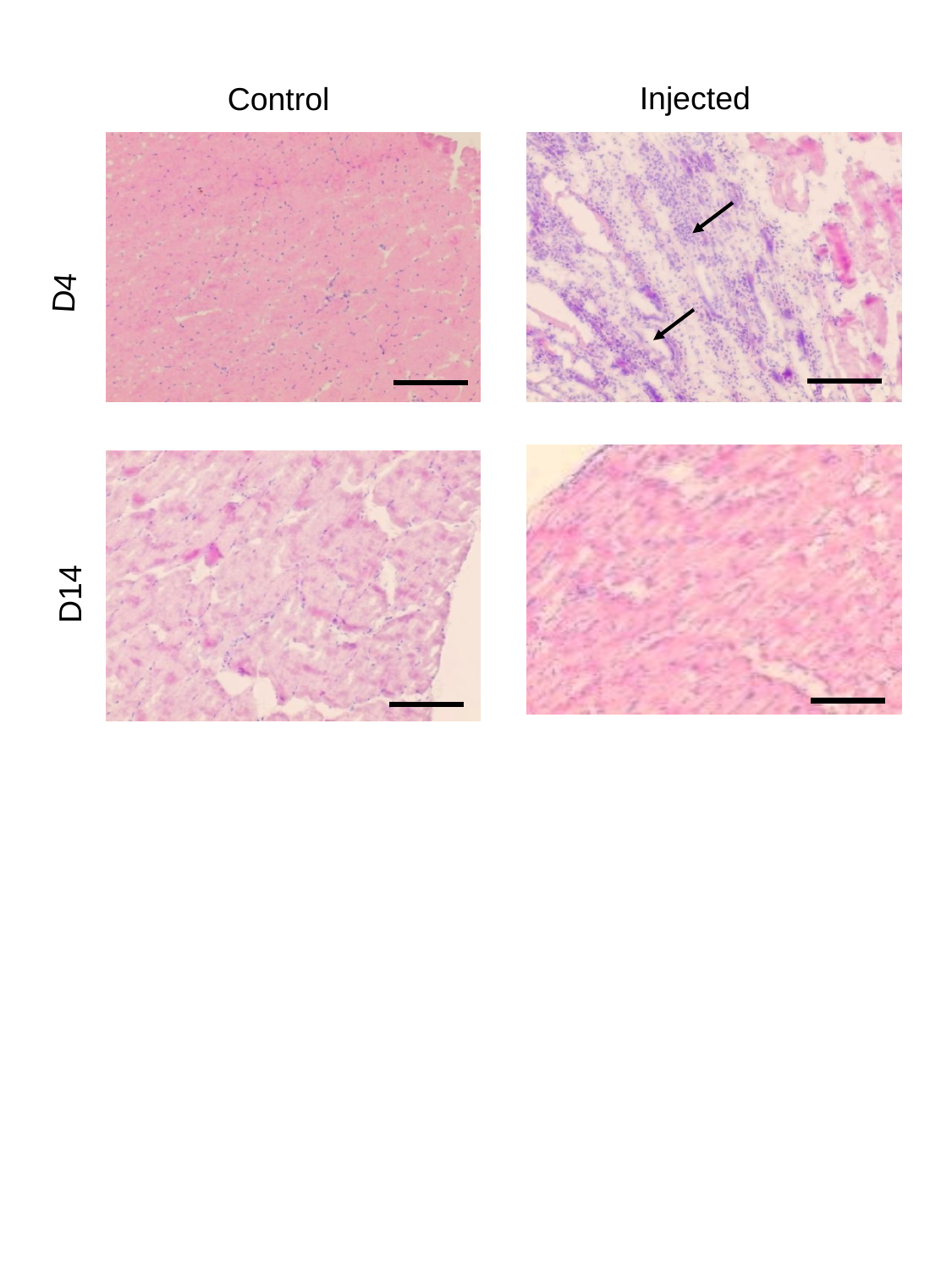

Injected
Control
D4
D14

## Slide 3
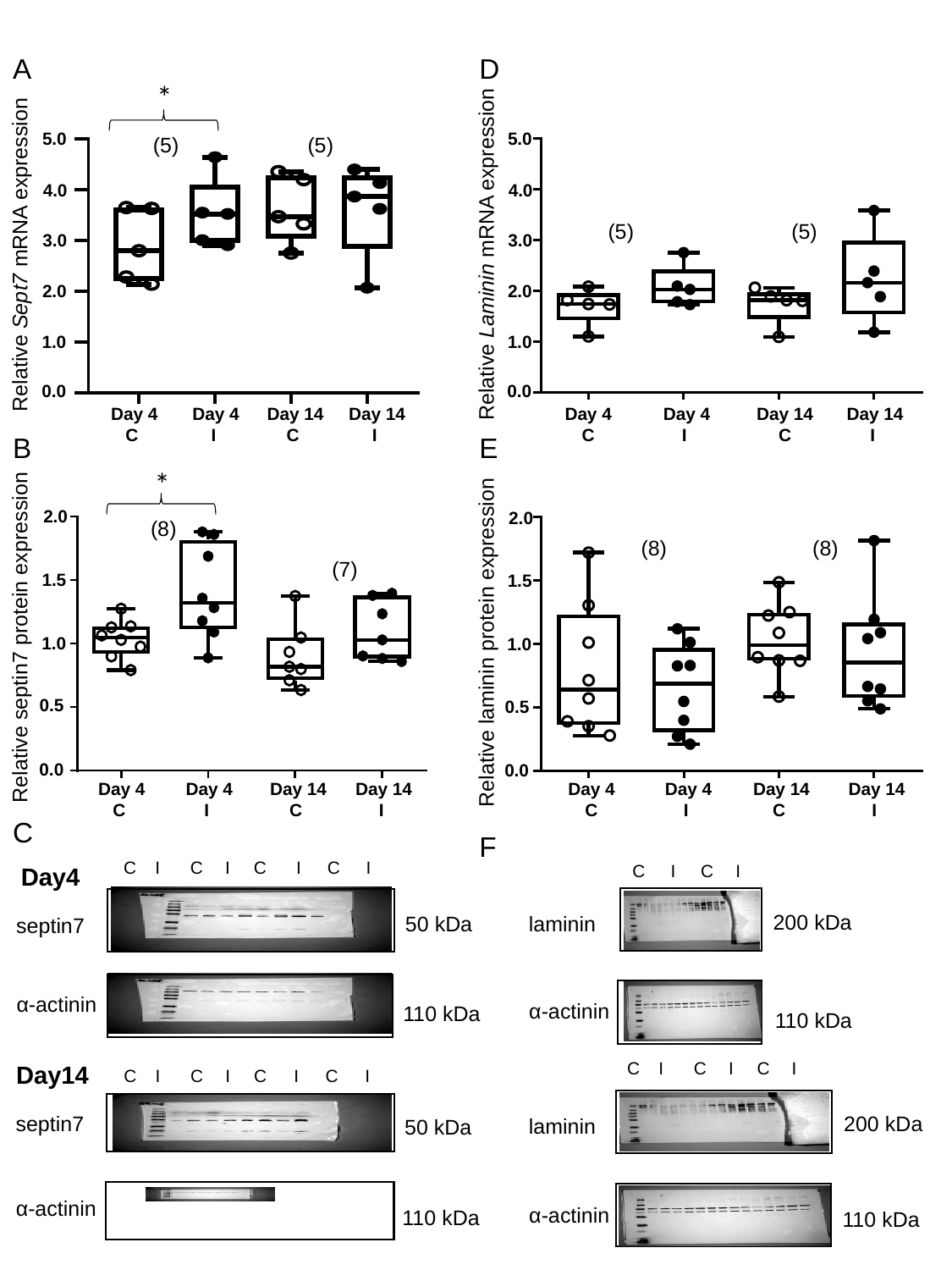

A
D
*
5.0
5.0
(5)
(5)
4.0
4.0
(5)
(5)
3.0
3.0
Relative Laminin mRNA expression
Relative Sept7 mRNA expression
2.0
2.0
1.0
1.0
0.0
0.0
Day 4
C
Day 4
I
Day 14
C
Day 14
I
Day 4
C
Day 4
I
Day 14
C
Day 14
I
B
E
*
2.0
2.0
(8)
(8)
(8)
(7)
1.5
1.5
Relative septin7 protein expression
Relative laminin protein expression
1.0
1.0
0.5
0.5
0.0
0.0
Day 4
C
Day 4
I
Day 14
C
Day 14
I
Day 4
C
Day 4
I
Day 14
C
Day 14
I
C
F
C
I
C
I
C
I
C
I
C
I
C
I
Day4
200 kDa
laminin
50 kDa
septin7
α-actinin
α-actinin
110 kDa
110 kDa
C
I
C
I
C
I
Day14
C
I
C
I
C
I
C
I
septin7
200 kDa
laminin
50 kDa
α-actinin
α-actinin
110 kDa
110 kDa

## Slide 4
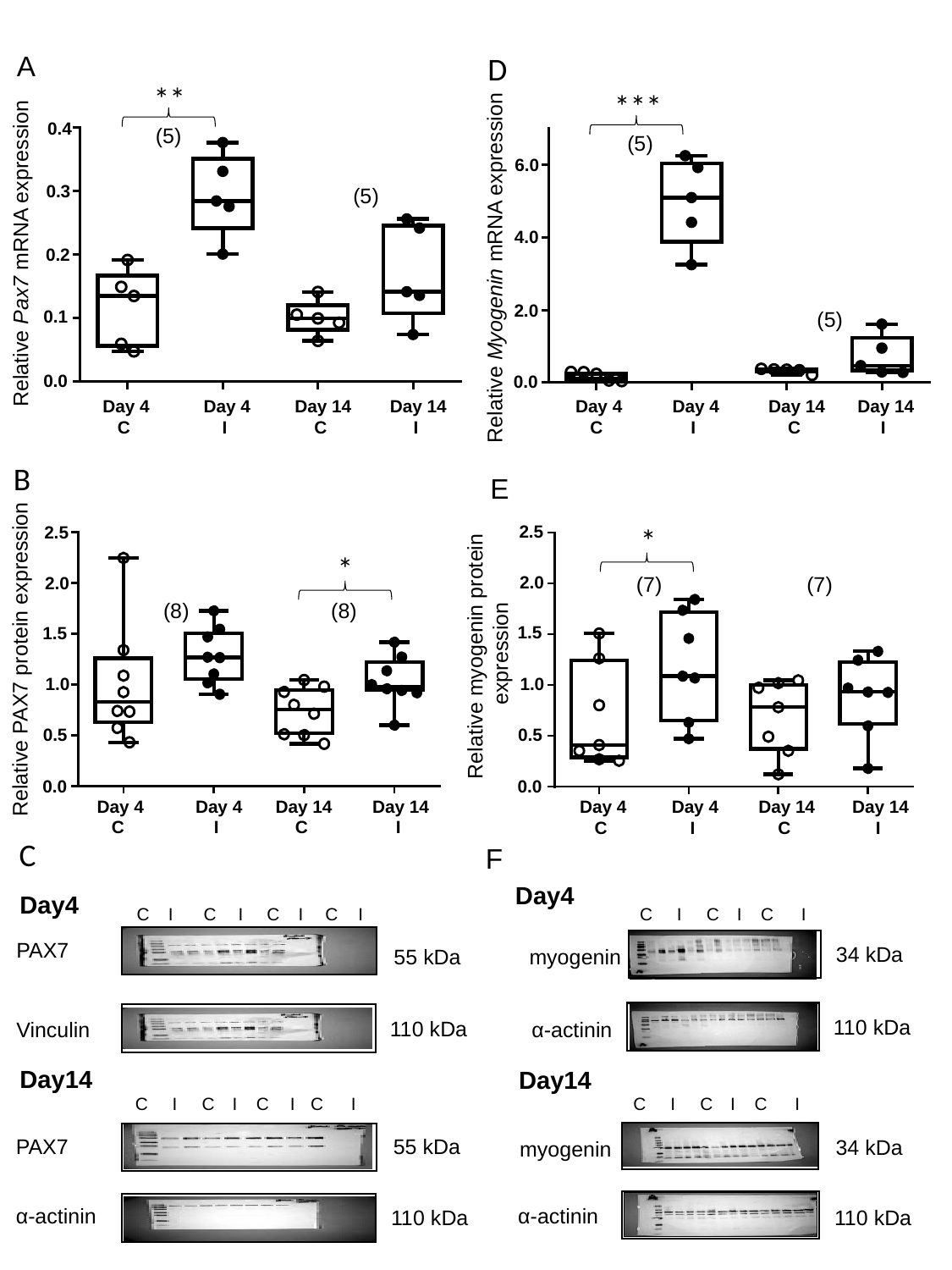

A
D
**
***
0.4
(5)
(5)
6.0
0.3
(5)
4.0
Relative Pax7 mRNA expression
0.2
Relative Myogenin mRNA expression
2.0
(5)
0.1
0.0
0.0
Day 4
C
Day 4
I
Day 14
C
Day 14
I
Day 4
C
Day 4
I
Day 14
C
Day 14
I
B
E
*
2.5
2.5
*
(7)
(7)
2.0
2.0
(8)
(8)
1.5
1.5
Relative myogenin protein
 expression
Relative PAX7 protein expression
1.0
1.0
0.5
0.5
0.0
0.0
Day 4
C
Day 4
I
Day 14
C
Day 14
I
Day 4
C
Day 4
I
Day 14
C
Day 14
I
C
F
Day4
Day4
C
I
C
I
C
I
C
I
C
I
C
I
C
I
PAX7
34 kDa
myogenin
55 kDa
110 kDa
110 kDa
Vinculin
α-actinin
Day14
Day14
C
I
C
I
C
I
C
I
C
I
C
I
C
I
55 kDa
PAX7
34 kDa
myogenin
α-actinin
α-actinin
110 kDa
110 kDa

## Slide 5
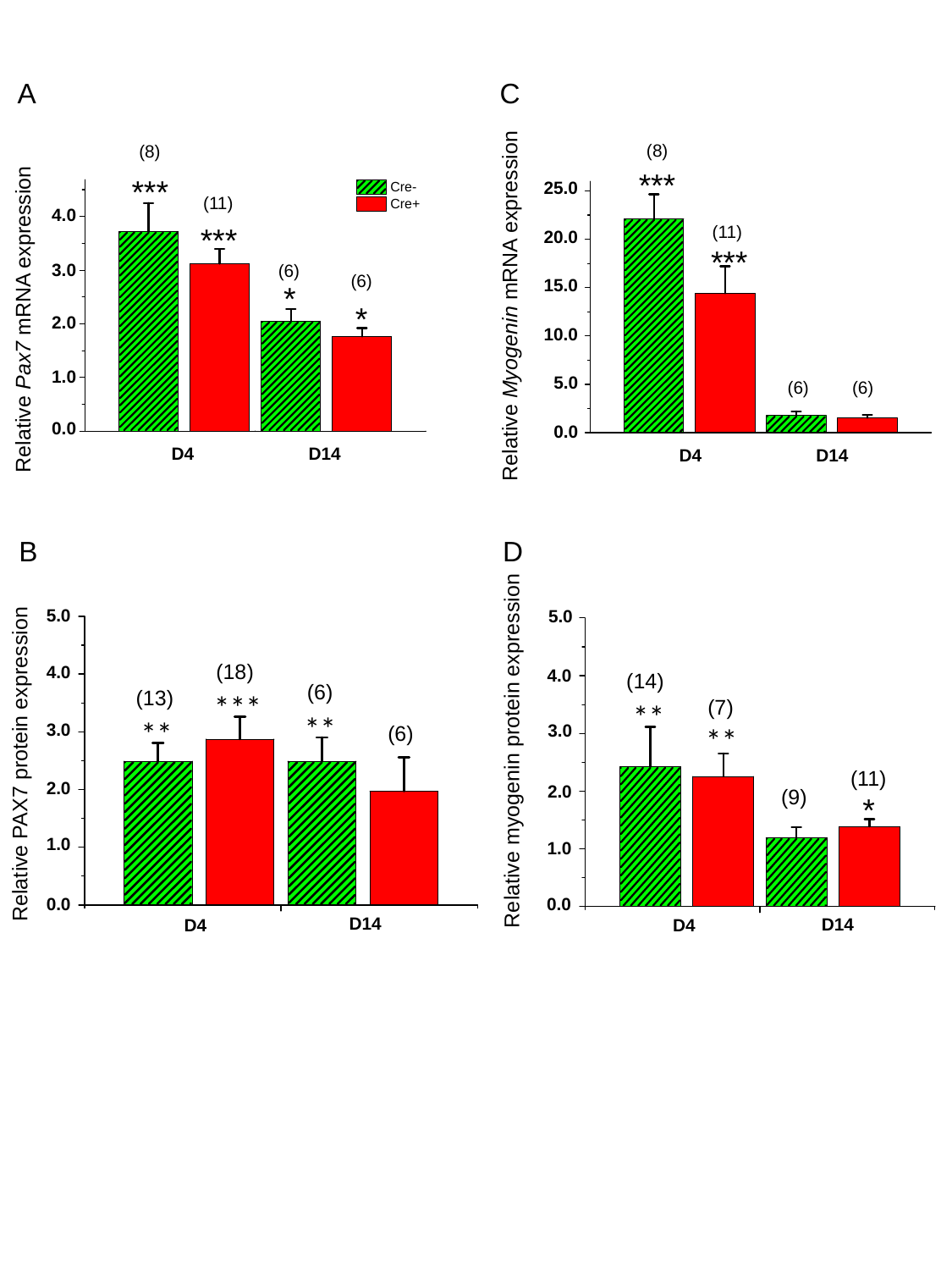

A
C
(8)
(8)
***
***
25.0
(11)
4.0
***
(11)
20.0
***
3.0
(6)
(6)
15.0
*
Relative Myogenin mRNA expression
*
Relative Pax7 mRNA expression
2.0
10.0
1.0
5.0
(6)
(6)
0.0
0.0
D14
D4
D14
D4
B
D
5.0
5.0
(18)
4.0
4.0
(14)
(6)
(13)
***
(7)
**
**
**
3.0
(6)
3.0
**
Relative myogenin protein expression
Relative PAX7 protein expression
(11)
2.0
2.0
(9)
*
1.0
1.0
0.0
0.0
D14
D14
D4
D4

## Slide 6
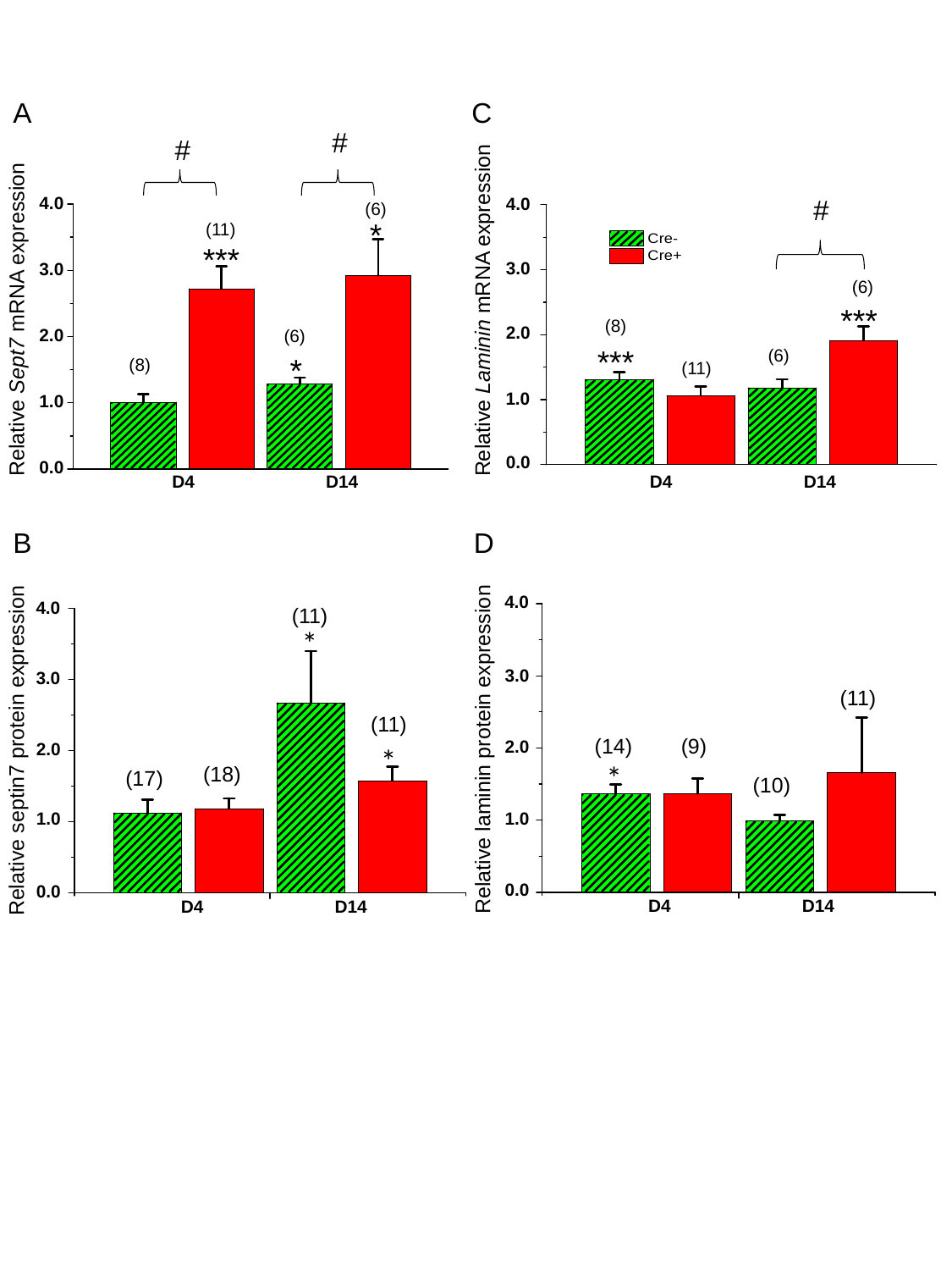

A
C
#
#
4.0
#
4.0
(6)
*
(11)
***
3.0
3.0
(6)
Relative Laminin mRNA expression
***
Relative Sept7 mRNA expression
(8)
2.0
2.0
(6)
***
(6)
*
(8)
(11)
1.0
1.0
0.0
0.0
D4
D14
D4
D14
B
D
4.0
4.0
(11)
*
3.0
3.0
(11)
(11)
(9)
(14)
2.0
Relative laminin protein expression
Relative septin7 protein expression
2.0
*
*
(18)
(17)
(10)
1.0
1.0
0.0
0.0
D4
D14
D4
D14

## Slide 7
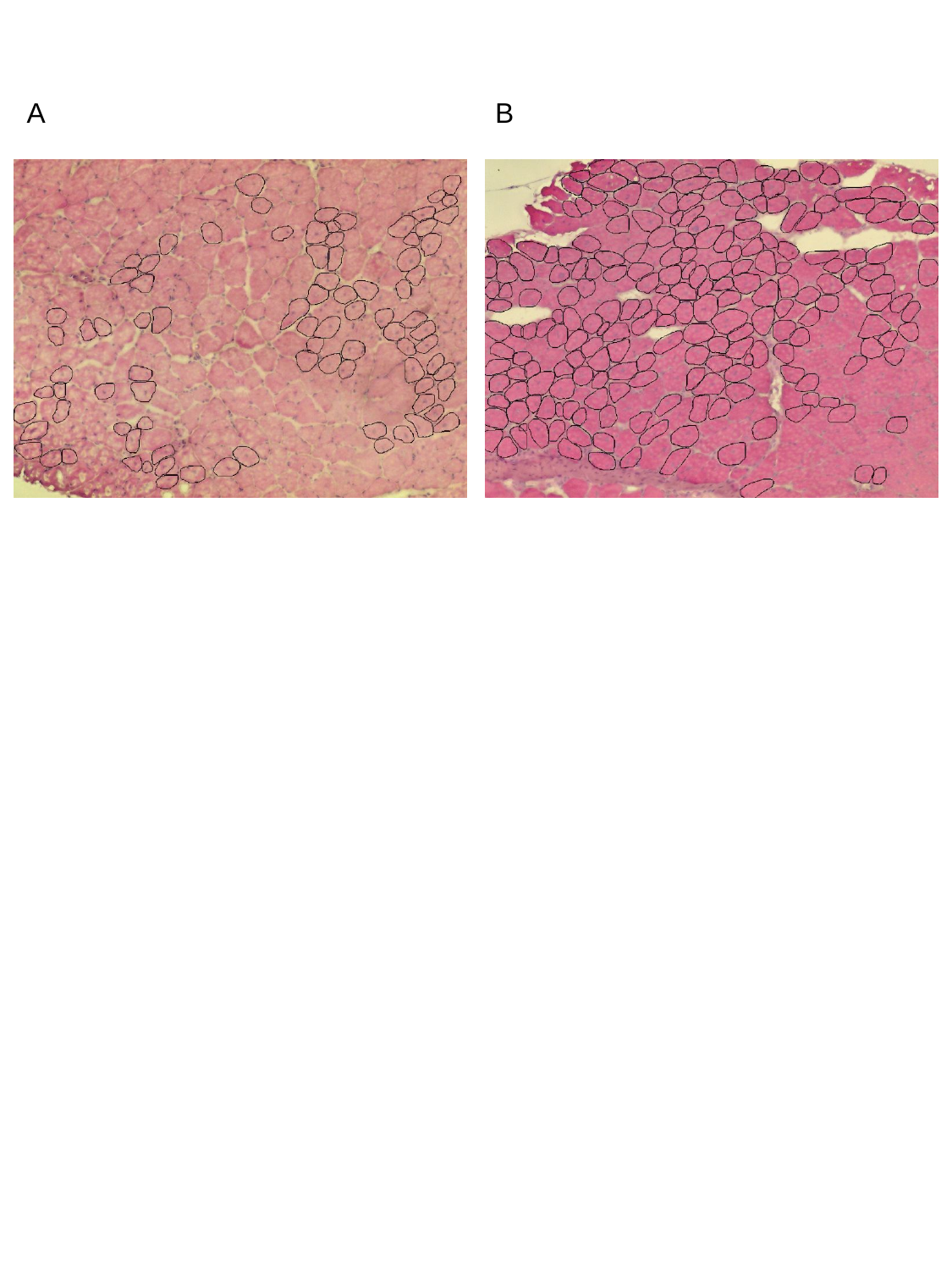

A
B

## Slide 8
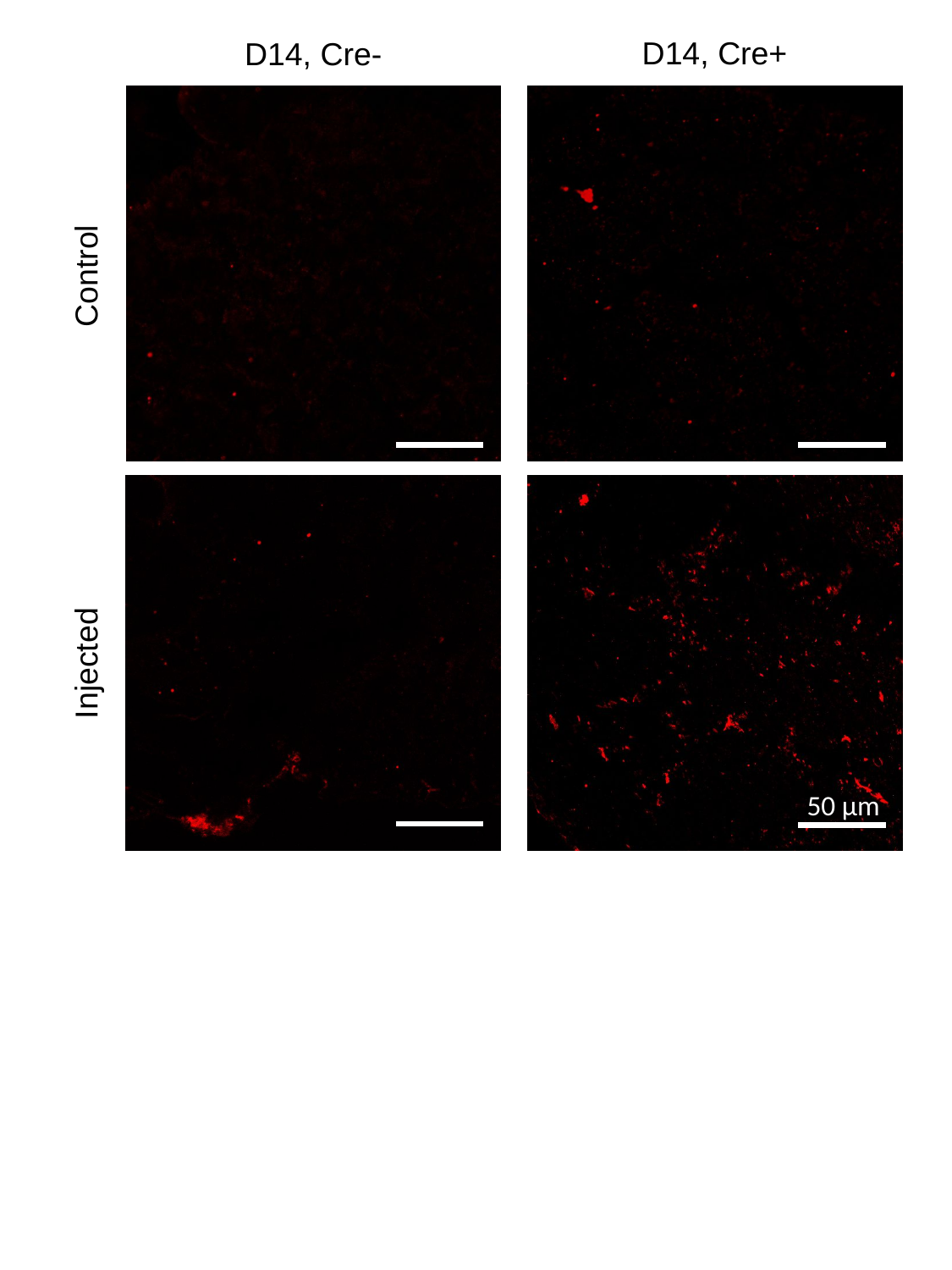

D14, Cre+
D14, Cre-
Control
Injected
50 μm
